# The efficacy of preoperative PET/CT for prediction of curability in surgery for locally advanced gastric carcinoma

**DOI:** 10.1186/1477-7819-8-86

**Published:** 2010-10-11

**Authors:** Hoon Hur, Sung Hoon Kim, Wook Kim, Kyo Young Song, Cho Hyun Park, Hae Myung Jeon

**Affiliations:** 1 Department of Surgery, Ajou University, School of Medicine, Suwon, Korea; 2 Department of Nuclear Medicine, The Catholic University of Korea, College of Medicine, Seoul, Korea; 3 Department of Surgery, The Catholic University of Korea, College of Medicine, Seoul, Korea

## Abstract

**Background:**

The benefits of preoperative ^18^FDG-PET/CT for gastric cancer remain uncertain. The aim of this study was to investigate the effects of preoperative ^18^FDG-PET/CT on the surgical strategy for locally advanced gastric cancer retrospectively.

**Methods:**

From January 2007 to November 2008, ^18^FDG-PET/CT was performed in 142 patients who had been diagnosed with advanced gastric cancer by computed tomography or gastrofiberscope findings.

**Results:**

Detection rates were 88.7% (126/142) for primary tumors and 24.6% (35/142) for local lymph nodes (LN). Nine patients with metastatic lesions underwent induction chemotherapy without operation. Of 133 patients subjected to operation, positive FDG uptake in primary tumors (*p *= 0.047) and local lymph nodes (*p *< 0.001) was related to non-curable operations. The mean standard uptake value (SUV) of primary tumors of patients who underwent non-curable operations was significantly higher than that of patients with curable operations (*p *= 0.001). When the SUV was greater than 5 and FDG uptake of LN was positive, non-curable operations were predicted with a sensitivity of 35.2%, a specificity of 91.0% and an accuracy of 76.7%.

**Conclusions:**

High SUV of the primary tumor and positive FDG uptake in local lymph nodes at PET/CT could predict non-curative resection in locally advanced gastric cancer. Therefore, information from preoperative PET/CT can help physician decisions regarding other modalities without laparotomy.

## Background

Preoperative imaging studies are used to evaluate clinical and surgical factors of malignant tumors, including resectability and identification of metastatic lesions that contraindicate resection. Although the presence of loco-regional disease in imaging studies will direct the surgical oncologist toward exploration with the intention of complete resection, the ability of these studies to exclude non-curability in surgery remains controversial.

In gastric cancer, the primary aim of surgery is curability, i.e., elimination of macroscopic and microscopic remnants of the malignant tumor by resection of the stomach and proper lymphadenectomy [1]. Since non-curative treatment is a definite poor prognostic factor for patients who undergo surgery for gastric cancer [2,3], other modalities may be needed in order to increase their survival. However, it is not easy to preoperatively diagnose non-curability by conventional non-invasive imaging methods such as computed tomography (CT), endoscopic ultrasound (EUS) and magnetic resonance imaging (MRI) without laparotomy or laparoscopic staging under general anesthesia.

Positron emission tomography (PET) imaging using the radiolabeled glucose analog ^18^fluorodeoxyglucose (FDG) can present biologic images according to glucose metabolism. PET imaging can be combined with anatomic imaging such as conventional CT scanning in order to increase diagnostic accuracy [4]. Although the National Comprehensive Cancer Network (NCCN) recently announced that preoperative PET/CT for gastric cancer patients can be recommended as an option of preoperative staging [5], the benefits of PET/CT remain uncertain.

Therefore, we analyzed information from preoperative PET/CT for patients with locally advanced gastric cancer and compared it with the surgical results, retrospectively. Uptake of FDG in the primary tumor or local lymph node and the standardized uptake value (SUV) were investigated for their potential in preoperative prediction of non-curative surgery. Thus, the aim of this study was to investigate the effects of preoperative PET/CT on the surgical strategy in gastric cancer patients.

## Methods

### Patient selection and study

From January 2007 to November 2008, our institution performed whole body ^18^F-FDG PET/CT scans for 142 consecutive patients about three days before surgery. These patients had been pathologically diagnosed with gastric adenocarcinoma by endoscopic biopsy and suspected of having advanced gastric cancer by endoscopic findings or conventional enhanced CT scans. They underwent careful physical examinations and other imaging studies such as bone scans and chest radiography in order to exclude distant metastasis. We obtained written informed consent from the patients for preoperative PET/CT, and then collected their preoperative staging data and surgical results for this retrospective study.

### PET/CT imaging

Before PET/CT scanning, all patients fasted for at least 6 hours. Patients were confirmed to have blood sugar levels below 130 mg/mL and rested for approximately 45 minutes before receiving an intravenous injection of 440 MBq of 18F-FDG. Scanning began 60 minutes later. A combined PET/CT in-line system (Biograph LSD, Siemens, Knoxville, TN) was used for all data collection. CT scanning was performed from the orbitomeatal line to the upper thigh (30 mA; 130 kV; 5 mm-thick sections) prior to PET. PET was then immediately conducted over the same body region with 6-8 bed positions, with 2 min acquisition time per bed position.

### Interpretation of PET/CT

PET/CT images were reviewed at a workstation with fusion software (Syngo, Siemens, Knoxville, TN) by a nuclear medicine physician who was given information about the clinical findings in the patient. The images were analyzed for the site and amount of positive FDG uptake; FDG uptake was defined as qualitatively positive when focal uptake was higher than normal background FDG activity in the primary tumor, local lymph node and metastatic lesions. FDG uptake in the bowel was regarded as positive when there was wall thickening of the same bowel at CT scan. The FDG uptake activity within each lesion was corrected by the administered dose and the patient weight to produce a maximum standardized uptake value (SUV). For this study, we only evaluated the SUV to primary tumors.

### Conventional CT scan

Conventional abdominal enhanced CT scanning (LightSpeed VCT, GE Healthcare, Milwaukee, WI) was performed after intravenous administration of contrast agents, with 5- to 10-mm slice thickness from the diaphragm to the symphysis pubis. The image was also reviewed by a radiologist who was provided with patient information. Non-curable operation was defined on CT scans when suspicious findings met the criterion of metastatic or non-resectable primary tumors in the surgical strategy.

### Treatment Plan

In our institution, we have the following treatment strategy for gastric cancer: patients who have metastatic lesions in either PET/CT or CT are started on induction chemotherapy with or without pathologic confirmation. Metastatic lesions of gastric cancer include liver and retroperitoneal lymph nodes or seeding into the peritoneum. A non-resectable primary tumor is indicated by pancreatic or duodenal invasion requiring pancreaticoduodenectomy, or invasion into the root of the meso-colon. Cases with only one modality of PET/CT and CT showing metastatic or non-resectable primary tumors undergo additional imaging studies such as magnetic resonance image (MRI) and ultrasound (US). Patients with suspicious metastatic lesions in the imaging study are subjected to surgical staging.

### Surgery

If the patient had suspicious metastatic lesions or a non-resectable primary tumor in the imaging studies, we first performed a minilaparotomy in order to confirm metastasis or the possibility of resectability. The abdominal incision was extended in cases with resectability in the surgical findings, and then surgery was performed by conventional open gastrectomy with over D1 plus beta lymphadenectomy with the intention of curability. Non-curable operation was defined when we performed open and close bypass surgery without tumor resection due to metastatic lesions in other organs, the peritoneum and retroperitoneal lymph, or when non-resectable primary tumors were found during surgery. In addition, palliative resection of primary tumors in which microscopic (R1) or macroscopic (R2) tumors remained was also included in the category of non-curative operation.

### Statistical analysis

Statistical analysis was performed with the statistical package for social sciences (SPSS) version 13.0. A Chi-square test was performed in order to evaluate differences of FDG uptake rates in primary tumors or local lymph nodes according to the clinico-pathological factors. The SUVs of curable and non-curable operations were compared by an independent t-test. The extent to which the SUV differed between a curable and non-curable operation was assessed using receiver operator characteristics (ROC) plots. We plotted ROC curves for SUV to predict non-curable operation, and then calculated sensitivity, specificity, accuracy and the positive predictive value at different SUV cutoffs (5, 7 and 9) as well as positive uptake of local lymph nodes.

## Results

In 142 enrolled patients, the FDG uptake rate of primary tumors was 88.7% (126/142) and that of local lymph nodes was 24.6% (32/142). The mean SUV of primary cancers was 5.7 (range, 1.89-19.06). In 2 patients, other simultaneous malignancies (thyroid cancer and rectal cancer) that the other imaging study could not detect were incidentally found. We performed combined operations for those patients.

**Figure 1:**
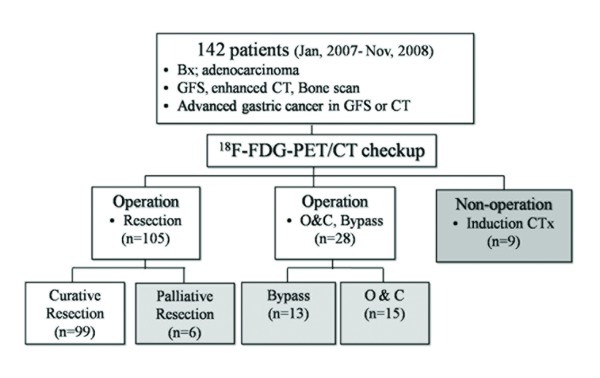
**Treatment strategies for patients diagnosed with gastric adenocarcinoma**. GFS = gastrofiberscopy, CT = computed tomography, O&C = open and closure, CTx = chemotherapy.

Nine patients who had metastatic lesions or non-resectable primary tumors in either PET/CT or conventional CT scan were not operated on. The PET/CT findings of these patients are listed in Table 1, showing that all patients had positive FDG uptake in the primary tumor. We performed operations on the remaining 133 patients and then evaluated the possibility of curative surgery (Fig 1).

**Table 1:** Study results of patients who underwent induction chemotherapy without operation

*No*	*CT finding*	*PET/CT finding*			*Additional study*
			
		Primary SUV	Local LN SUV	Other uptake	
1	Lung metastasis	2.97	2.97	Lung, Bone	Spine MRI
2	Peritoneal seeding Liver metastasis	6.81	5.44	Mesentery	
3	Peritoneal seeding Esophagus invasion	7.89	0	Distal Esophagus	
4	Peritoneal seeding	3.91	3.34	Peritoneum	
5	Peritoneal seeding	3.73	8.15	Retroperitoneal LN Lt. supraclaviclar LN	
6	Liver metastasis	10.73	0	Liver	Sono, Liver MRI
7	Liver metastasis	7.26	12.18	Liver	Sono
8	Peritoneal seeding	2.4	0	T-colon, Omentum, Retroperitoneal LN	
9	Liver metastasis	11.9	0	Liver	

PET, positron emission tomography, CT, computed tomography, LN, lymph nodes, SUV, standardized uptake value, MRI, magnetic resonance imaging

The clinico-pathological characteristics of the 133 patients who underwent surgery are presented in Table 2. The rates of FDG uptake in the primary tumor and local lymph nodes were compared according to age, gender, diabetic mellitus, tumor size, tumor location, histology and curability of operations. Except for non-curative operation (97.1% vs. 84.8%, *p *= 0.047), no factors were significantly correlated with the FDG uptake rate in the primary tumor. Patients with large tumor sizes showed relatively high uptake rates in the primary tumor (92.6% vs. 83.1%, *p *= 0.090). The FDG uptake rate of local lymph nodes was significantly higher in patients who underwent non-curative operations (44.1% vs. 14.1%, *p *< 0.001).

**Table 2:** Preoperative and operative findings of PET/CT in patients who underwent operation (n = 133)

		*n*	*FDG uptake in primary tumor*			*FDG uptake in local LN*		
				
			*Yes (%)* *(n = 117)*	*No (%)* *(n = 16)*	*p-value*	*Yes (%)* *(n = 29)*	*No (%)* *(n = 104)*	*p-value*
Age(years)	< 60	53	46(86.8)	7(12.1)	0.734	11(20.8)	42(79.2)	0.811
	≥ 60	80	71(88.8)	9(10.7)		18(22.5)	62(77.5)	
								
Gender	Male	92	82(89.1)	10(10.9)	0.570	22(23.9)	70(76.1)	0.378
	Female	41	35(85.4)	6(14.6)		7(17.1)	34(82.9)	
								
DM	Positive	18	16(88.9)	2(11.1)	1.000	5(27.8)	13(72.2)	0.543
	Negative	115	101(87.8)	14(12.2)		24(20.9)	91(79.1)	
								
Size(cm)	< 5	65	54(83.1)	11(16.9)	0.090	10(15.4)	55(84.6)	0.080
	≥ 5	68	63(92.6)	5(7.4)		19(27.9)	49(72.1)	
								
Location	Upper	22	20(90.9)	2(9.1)	1.000	5(22.7)	17(77.3)	0.909
	Middle and lower	111	97(87.4)	14(12.6)		24(21.6)	87(78.4)	
								
Histology	Tubular carcinoma	108	95(88.0)	13(12.0)	1.000	25(23.1)	83(76.9)	0.435
	Signet ring/mucinous	25	22(88.0)	3(12.0)		4(16.0)	21(84.0)	
								
Curability	Curative operation	99	84(84.8)	15(15.2)	0.047	14(14.1)	85(85.9)	< 0.001
	Non-curative operation	34	33(97.1)	1(2.9)		15(44.1)	19(55.9)	

PET, positron emission tomography, CT, computed tomography, FDG, fluorodeoxyglucose, LN, lymph nodes, DM, diabetes mellitus

**Figure 2:**
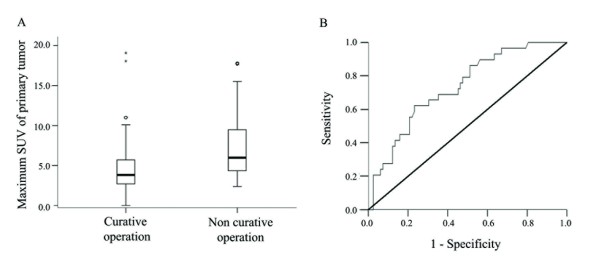
**Maximum SUV of primary tumor related to curative or non-curative operation**. A: Box plot of maximum SUV of primary tumor in patients with curative or non-curative surgery; the mean values were significantly different between the two groups in an independent t-test (*p *< 0.001). **B: **Receiver operator characteristics (ROC) curve of maximum SUV of primary tumor for predicting non-curative operation. The area under the curve was 0.730 (*p *< 0.001, 0.629 <95% C.I. <0.831).

The mean maximum SUV of primary tumors in patients with non-curative operations was 7.3 ± 4.5 (mean ± S.D.) and that of patients with curative operations was 4.4 ± 3.5 (mean ± S.D.). The difference in SUV between the two groups was significant (p = 0.001), and a box plot of the SUVs in both groups is presented in Fig 2A.

An ROC curve of the maximum SUV was plotted in order to predict non-curative operations, and an area under the curve of 0.730 (*p *< 0.001; 0.629 < 95% C.I. < 0.831) was obtained (Fig 2B). We calculated diagnostic indices (sensitivity, specificity, accuracy and positive predictive value) at various SUV cutoffs for primary tumor and lymph node FDG uptake, and then compared these results with predictions from conventional enhanced CT scanning. When the maximum SUV was greater than 5 and the FDG uptake of lymph node was positive, non-curative operation was predicted with a sensitivity of 35.2%, a specificity of 91.0%, an accuracy of 76.7% and a positive predictive value of 57.1%. These values are higher than those obtained using other SUV cutoffs for primary tumors or even with conventional enhanced CT scanning (sensitivity of 17.6%, specificity of 87.9%, accuracy of 69.9% and a positive predictive value of 33.3%) (Table 3).

**Table 3:** Prediction of non-curative operation in patients who underwent operation (n = 133)

	*n*	*Sensitivity*	*Specificity*	*Accuracy*	*Positive predictive value*
Enhanced CT Scan (Suspicious non-curability)	18	0.176	0.879	0.699	0.333
Tumor SUV > 5	54	0.676	0.687	0.684	0.426
Tumor SUV > 7	24	0.353	0.879	0.744	0.500
Tumor SUV > 9	17	0.265	0.919	0.752	0.530
Local LN SUV uptake (+)	29	0.441	0.859	0.752	0.517
SUV > 5 and LN (+)	21	0.352	0.910	0.767	0.571

CT, computed tomography, SUV, standardized uptake value, LN, lymph nodes

## Discussion

For patients with locally advanced gastric cancer, the preoperative prediction of curability is important because it can prevent unnecessary laparotomies and direct physicians toward treatment with other modalities such as neoadjuvant chemotherapy. Conventional enhanced CT scans are one of the most important imaging methods for preoperative prediction of curability. Therefore, patients diagnosed with definite metastatic lesions (cM1) by CT scan might be treated systemically without surgery. However, the treatment strategy for patients with locally advanced gastric cancer and without definite cM1 lesions has often been decided based on surgical findings following laparotomy or laparoscopic staging [6]. Our results in patients with locally advanced gastric cancer show that preoperative ^18^F-FDG PET/CT could provide objective information for decisions regarding treatment strategies such as laparoscopic staging and neoadjuvant chemotherapy.

At present, several studies have reported that FDG-PET is the most sensitive non-invasive imaging strategy for detecting distant metastasis [7,8]. Therefore, our study was also designed that patients with suspected metastatic lesions on CT scanning accompanied by FDG uptake were started on induction chemotherapy without operation. Previous studies reported that FDG-PET, and not PET/CT, was more sensitive than CT scanning for detecting primary tumors in advanced disease, but inferior to CT for detecting intra-abdominal lymph node metastasis [8,9]. In addition, recent studies showed that FDG-PET had lower sensitivity for detection of lymph nodes metastasis, and even had no definite role as preoperative imaging in gastric cancer [10,11]. Moreover, studies validating the use of PET/CT in gastric carcinoma are lacking thus far, and most physicians cannot confirm whether adding CT information to FDG-PET will improve diagnostic accuracy. Due to these reasons, the current aims of preoperative PET/CT in most centers that perform operations for gastric cancer patients, including our institution, are as follows: 1) to confirm metastasis by contrast-enhanced CT scan; 2) to investigate metastatic lesions that are not detected by contrast-enhanced CT scan; 3) to evaluate other hidden simultaneous malignancies that are asymptomatic and undetectable by CT scanning. Contrary to above usage of PET-CT in gastric cancer, we focused on the prediction of surgical finding through the result of preoperative PET-CT. The results of our study suggested that treatment strategy of gastric cancer could be decided according to finding of FDG-PET CT.

With respect to preoperative PET/CT as a tool for surgical strategy decisions, the present study uncovered several relevant results. Using the semi-quantitative feature of FDG-PET/CT, the degree of FDG uptake of the primary tumor and the SUV was analyzed for prediction of curability. The mean SUV of the primary tumor in patients who underwent non-curative surgery was significantly higher than that of patients with curative surgery. Therefore, the SUV of the primary tumor might be a predictive factor for non-curative surgery; this is supported by the results of the ROC curve. When we defined a mean primary tumor SUV of greater than 5.0 and positive uptake of FDG in perigastric lymph nodes as cutoff values for prediction of non-curative resection, the sensitivity, specificity and accuracy were higher than those of enhanced CT scanning. Therefore, we find that FDG-PET/CT may be a tool for decisions concerning laparoscopic staging or neoadjuvant chemotherapy.

SUV values are common indices of tracer uptake in studies with PET, and can be calculated from the radioactivity of tumors following injection of fluorine ^18^F-FDG according to body weight and physical decay [12]. The possibility of applying the SUV to preoperative PET/CT as a predictor for curability is explained by the following. The SUV may represent the growth rate of malignant tumors. Several reports have described that glucose utilization is higher in rapidly growing tumors than in less aggressive neoplasia [13,14]. In our study, the mean SUV was correlated with curability of advanced gastric cancer.

Diagnostic laparoscopy for the staging of gastric cancer has the benefit for diagnosis of radiographically occult metastatic disease. However, laparoscopic staging requires general anesthesia and many studies have reported that most patients who undergo laparoscopic staging also have to undergo laparotomy [15-17]. In addition, animal studies have shown that pneumoperitoneum due to laparoscopic examination could impair immunity and promote tumor growth [18-20]. Therefore, the routine use of laparoscopic staging for patients with advanced gastric cancer has been questioned. Several studies have recommended that laparoscopic staging be performed in patients with advanced primary tumors (overinvasion into muscle propria) and no significant metastatic lesion, and avoided if the tumor does not involve the gastroesophageal junction and lymph node metastasis is absent on spiral CT or endoscopy ultrasound (EUS) [6,21]. However, the results of CT or EUS are frequently subjective depending on the radiologist or endoscopist, whereas PET/CT can establish objective information such as the uptake of FDG in primary tumors or lymph nodes and the degree of uptake presented as the SUV.

In terms of FDG uptake in local lymph nodes, although PET/CT added anatomical information of lymph node enlargement, PET scanning is limited in its ability to separate a local lymph node from a primary tumor due to intense tracer accumulation and ill-defined anatomical boundaries [22]. Metastatic local lymph nodes were identified by PET/CT when there were enlarged lymph node lesions with FDG uptake occurring separately from primary tumors. In addition, there enlarged or conglomerated lymph nodes can lead to unresectablilty due to the invasion of the metastatic nodes into the pancreas and major vessels like hepatic artery or celiac trunk. Therefore, although the positive rate of metastatic lymph nodes in PET/CT is not high, it may indicate as aggressive as these gastric cancers are difficult to cure with resection. In our study, positive lymph node metastasis in PET/CT was related to non-curative surgery; this might have higher predictive accuracy for non-curative surgery that the SUV of the primary tumor alone.

Our study has several limitations. First, the number of enrolled patients might be too small to confirm the clinical validity of PET/CT for gastric cancer. Therefore, studies enrolling larger populations should be planned in order to confirm the correlation between preoperative PET/CT and operative findings. Second, the criteria for non-curative surgery might be subjective. In this study, gastric cancer with definite distant metastatic lesions (M1) or with surgical findings of invasion into the pancreatic head were necessarily defined as non-curative surgery. Pancreaticoduodenectomy as a curative surgery for pancreatic invasions of gastric cancer requiring are controversial due to high operative morbidity and mortality [23,24]. Moreover, no results from clinical trials have confirmed the benefit of pancreaticoduodenectomy for gastric cancer. Third, although previous studies have reported a difference in FDG uptake rate according to the histological type of gastric cancer [9,25], this was not observed in our study. We believe that confining our enrollment of patients to those with advanced gastric cancer might mask the difference in FDG uptake by histological type, since the tumor size and depth of invasion can effect on the FDG uptake [9].

## Conclusions

Despite these limitations, our results show that high FDG uptake rate of the primary tumor and local lymph nodes is related to non-curable surgery. High SUV of the primary tumor and positive FDG uptake of local lymph nodes in PET/CT could predict non-curable surgery in locally advanced gastric cancer with higher specificity, accuracy and positive predictive values than those achieved by CT scan. Therefore, we suggest that gastric cancer patients showing high SUV in the primary tumor and positive FDG uptake in local lymph nodes at PET/CT should be subjected to neoadjuvant chemotherapy or laparoscopic staging in order to avoid unnecessary laparotomy. Furthermore, we will evaluate the correlation between preoperative PET/CT and post-operative prognosis through follow-up of the enrolled patients to enhance the clinical benefit of PET/CT.

## Competing interests

The authors declare that they have no competing interests.

## Authors' contributions

HH: Analysis of the data and drafting of the manuscript. SHK: Interpretation of data. WK, KYS, CHP: Revise it critically for important intellectual content. HMJ: Concept and design of the manuscript. All authors read and approved the final manuscript.
